# Supplementary material for: Association of reduced serum EGF and leptin levels with the pathophysiology of major depressive disorder: A case-control study
Source: PLoS One. 2023 Jul 3;18(7):e0288159. doi: 10.1371/journal.pone.0288159 (PMC10317226; doi:10.1371/journal.pone.0288159)
Supplement: Attachment — Submitted filename: Response to Reviewers.docx [file pone.0288159.s001.docx]

Dear Editors and Reviewers,

Thank you for your letter and the reviewers' comments on our manuscript entitled "Reduced serum EGF but not leptin levels are associated with the pathophysiology of major depressive disorder: A case-control study" (Manuscript ID PONE-D-23-05701). All the comments were valuable and helpful to the revision and improvement of the manuscript. We have carefully studied the comments and made corrections, which we hope will merit your approval. We marked the revised portions using track changes. Our point-by-point answers to the reviewers’ comments appear at the end of this letter.

We earnestly appreciate the Editors'/Reviewers' work. We hope that after this revision, the paper will be deemed fit for publication. We would be glad to respond to any further questions and comments that you may have.

Once again, thank you very much for your comments and suggestions.

Best regards,

**Md. Rabiul Islam, PhD**

Associate Professor, Department of Pharmacy, University of Asia Pacific

74/A Green Road, Farmgate, Dhaka-1205, Bangladesh.

Email: robi.ayaan@gmail.com; Cell: +8801916031831; ORCID iD: https://orcid.org/0000-0003-2820-3144

**Point by point authors’ responses to the reviewers**

Manuscript ID PONE-D-23-05701

*Title: Reduced serum EGF but not leptin levels are associated with the pathophysiology of major depressive disorder: A case-control study*

**Reviewer #1**

The manuscript is developed with an interesting subject.

***Author responses***

*Thank you for your review and observation. We appreciate your encouraging comments on our manuscript.*

**Comment:**

Lots of Acronyms used without elaboration. In title the acronym should be avoided. The abstract start with MDD is not understanding for many of the readers

***Author responses***

*Thank you for your observation. We have elaborated the acronym as per your suggestion (page 2, line 3, 12 & 16).*

**Comment:**

In abstract the objective is not clearly mentioned

***Author responses***

*Thank you for your observation. We have stated our objective more clearly (page 2, line 6-9).*

**Comment:**

The result section is very small, need to be elaborated.

***Author responses***

*Thank you for your suggestion. The result section has been elaborated.*

**Comment:**

The Odds Ratio (OR) is not analysed in the manuscript, but for case control study it is essential.

***Author responses***

*Thank you for your suggestion. We have analyzed our data using independent sample t-test and chi-square test were applied to differentiate between the groups for variables. Also, we performed several statistical tests to visualize the findings. It would be better we could perform Odds Ratio analysis for our dataset; however, it is difficult to accommodate this analysis at this stage. Thank you again for your observation.*

**Reviewer #2**

Congratulations on your hard work. However, I have some observations that may help with your manuscript.

***Author responses***

*Thank you for your review and observation. We appreciate your encouraging comments on our manuscript.*

**Comment 1:**

Title: you have stated the result in the title. I might suggest to keep it minimum, like- Association of Reduced serum EGF and leptin levels with the pathophysiology of major depressive disorder: A case-control study.

***Author responses***

*Thank you for your suggestion. We have revised the title following your suggestion.*

**Comment 2:**

Abstract, background, line 3: please rephrase the line: this study, we compared the serum levels of 'pro-inflammatory cytokine leptin and neurotrophic factor EGF' of healthy controls (HCs) and MDD patients.

***Author responses***

*Thank you for your suggestion. We have rephrased the line (page2, line 6-8).*

**Comment 3:**

Abstract, and background: please avoid abbreviations in the abstract. Also state the full term of MDD in the first line of background.

***Author responses***

*Thank you for your observation. We have stated the full form of MDD and also elaborated other acronyms in abstract.*

**Comment 4:**

Rephrase the line: On the one hand, trauma regarding 'physical, emotional, or sexual abuse' during childhood might also be a contributing factor to MDD.

***Author responses***

*Thank you for your suggestion. We have rephrased the line (page 3, line 15-16).*

**Comment 5:**

Please add some prevalence of MDD in Bangladeshi background also.

***Author responses***

*Thank you for your suggestion. The prevalence of depressive disorder in Bangladesh has been added (page 3, line 27-28).*

**Comment 6:**

In background, add the rationale of exploring EGF and leptin level in MDD in practical purpose. Will they help in the treatment of MDD?

***Author responses***

*Thank you for your observation and suggestion. We stated the reasons of exploring leptin and EGF levels and how it will help in understanding MDD (page 5, line 11-17).*

**Comment 7:**

Methods: State the hypothesis if you have any.

***Author responses***

*Thank you for your observation and suggestion. We have mentioned the hypothesis of our study in the introduction part (page 5, line 11-17).*

*It might be an option to state the hypothesis in the method section. But the way we designed our manuscript, we think the hypothesis is better fitted in the introduction section.*

**Comment 8:**

Methods: add brief description about HAM-D scale. How it is calculated/scored.

***Author responses***

*Thank you for your suggestion. We have added a short description about HAM-D scale (page 6, line 6-11).*

**Comment 9:**

Discussion: state the possible practical application of your finding at the end of discussion.

***Author responses***

*Thank you for your observation. The practical application of our findings has been added (page 10, line 6-8).*

**Comment 10:**

Table 1 & 2: state the statistical tests used to determine the p value under the tables.

***Author responses***

*Thank you for your suggestion. We have mentioned the respected tests used to determine the p-value under the tables.*

**Reviewer #3**

Thank you for inviting me to review the research paper titled “Reduced serum EGF but not leptin levels are associated with the pathophysiology of major depressive disorder: A case-control stud.”

I am writing to share my accepting of this study on the pathophysiology of depression. The research sheds important light on the complex mechanisms underlying depression and makes a significant contribution to the field. Even though the findings are still controversial and inconclusive yet but I commend the authors on the scientific rigor and meticulousness with which they presented the discussion, conclusions, recommendations, and limitations. Their attention to detail and thoughtful analysis demonstrates a deep understanding of the complexities of depression and reflect noted expertise and commitment to advancing the field. These findings have the potential to help future research and improve our understanding of depression, ultimately leading to better treatments and outcomes for those affected.

Well done.

***Author responses***

*Thank you for your review and valuable observation. We appreciate your encouraging statement about our manuscript.*

**Reviewer #4**

The current manuscript aims to investigate whether the EGF and Leptin serum levels differ in MDD group from HCs. While I think that the research idea is very interesting, some changes seem to be essential for improving the academic quality of manuscript. Please see my comments below. Anyway, be it for resubmission in this journal or a submission to a different journal, I think the manuscript can be strengthened on condition of major revisions concerning all parts of the manuscript. I wish the authors the best of luck with this and their future research.

***Author responses***

*Thank you for your effort to review our manuscript. We appreciate your encouraging comments on our manuscript.*

***Comment on Introduction***

**Comment 1:**

The writing style of Reference 1,2,3. etc. seem not to meet to reference rules. (All references need to be reviewed).

***Author responses***

*Thank you for your observation. We have reviewed the entire manuscript and rewrite the references where necessary.*

**Comment 2:**

Many of the cited articles are rather old and there is newer evidence that should be considered and given the credit it deserves, for instance 10, 21-24, 25, 27-29, 31 (I strongly recommend that the references be reviewed for currency).

***Author responses***

*Thank you for your observation. We have added the recent published articles as references instead of the old ones where necessary.*

**Comment 3:**

“According to a survey report, the prevalence of MDD in a calendar year varied by country, which is about 2.20% and 10.40% in Japan and Brazil, respectively. Another study has revealed that people from middle-income nations are more affected by MDD than people from high-income nations.”
“All of those hypotheses are supported by investigations regarding mental stress and findings about an increase in the level of hormones released due to stress like a CRH (corticotropin-releasing hormone), reduction in the availability of neurotransmitters, e.g., dopamine, noradrenaline, and serotonin in the synaptic cleft, alteration in the levels of inhibitory and excitatory neurotransmitters such as GABA, glutamate, respectively.”

can you provide a reference supporting these claims?

***Author responses***

*Thank you for your observation and suggestion. The statements have been modified and references have been added accordingly. You will find this information in page 3, line 22-27; page 4, line 1-6.*

**Comment 4:**

I think that the authors did not provide enough rationale for why they focused on EGF and Leptin together. As far as it is understood from the last 2 paragraphs, they preferred to emphasize only discrepancy, but the presence of contradictions in the relationship between pro- and anti-inflammatory parameters and depression in general seems to make this background somewhat inadequate. Indeed, to the extent that it is essential to consider the two parameters together, the authors should propose a solution to the problem of multiple comparisons (type-II error) in the analyses in which they are included as dependent variables. The section on the epidemiology of depression in the first half seems to overshadow the intended message to some extent. Instead, I would have preferred to expand the framework on why Leptin and EGF were included together, and a separate paragraph on the hypotheses would have made it easier for readers. If the authors take this route, I believe that whether they present their hypotheses on Leptin and EGF as the same or separately will influence the results of the study.

***Author responses***

*Thank you for your observation and suggestion. We have tried to elaborately describe the rationality of performing this study as per your suggestion. You will find the respected information in page 5, line 11-17.*

***Comment on Method***

**Comment 1:**

The authors should indicate which treatments they excluded with the statement "Patients undergoing any therapy that could affect the concentration of serum leptin and EGF levels". For example, if they included the cases under antidepressant treatments, is the association with these two parameters at a level that requires to be considered as a control factor? Adding the basics of these to the "Introduction" would have complemented the biological framework of the article.

***Author responses***

*Thank you for your observation. We have rephrased the statement (page 6, line 1-3). In our study, we ensured that same exclusion criteria were maintained for all participants.*

***Comment on Results***

**Comment 1:**

Table 2 shows that among male participants, serum EGF levels did not differ statistically from HC in MDD patients. The authors seem to have no indication of a gender effect, which I recommend controlling for in a model.

***Author responses***

*Thank you for your observation and suggestion. We have tried to explore the serum concentration differences between MDD and control subjects to explore the gender variability among our participants after laboratory analysis. This might help in exploring the association of leptin and EGF with depression between males and females.*

*At the same time, the independent sample t-test was performed between MDD male and MDD female subjects to find out the differences. But no significant differences were observed.*

**Comment 2:**

The information displayed in Table 1 seems to be (at least) redundant to the text in the Results part. Please decide whether you want to report it either in the main text, or in the table, but not both.

***Author responses***

*Thank you for your observation and suggestion. As per your other comment, the information displayed in Table 1 was summarized (page 7, line 21-26).*

**Comment 3:**

Adding correlation slopes in the scatter plot would make the visualization more meaningful. Also, I don't think the scatter plot is the appropriate graph to understand the “more severe depression levels of female MDDs”. Presenting the correlation coefficients in the text or in the graph will achieve the main goal here.

***Author responses***

*Thank you for your observation and suggestion. We have removed our statement and changed the scatter plot as per your suggestion.*

**Comment 4:**

In addition, the characteristics of the study population are still given more space than the main results, and if they are to be presented in the text, it is more appropriate to summarize them. Instead, I suggest adding the points I mentioned to the main results.

***Author responses***

*Thank you for your observation. The information displayed in Table 1 was summarized (page 7, line 21-26).*

***Comment on Discussion***

I assume that there might be a few changes to the discussion part based on my comments on the other parts, so I refrain from repeating my previously stated thoughts for all other parts of the manuscript and just focus on some additional comments I had for preparing a revision.

***Author responses***

*Thank you for your effort to review our manuscript.*

**Comment 1:**

In the first paragraph, the authors begin the discussion section with a theoretical background that they have already included in the introduction. This seems to cause some disconnection in the narrative flow. Instead, I would suggest that the second paragraph should be placed first and the framework in the first paragraph should still be used in other parts of the discussion if necessary. I also think it is important that they state the extent to which the hypotheses I suggest to be presented in the introduction have been confirmed.

***Author responses***

*Thank you for your observation and suggestion. We have modified our manuscript and omitted the mentioned first paragraph as per your suggestion.*

**Comment 2:**

The statements in paragraph 3 “On the one hand, in this study, we also measured the serum levels of EGF in MDD patients compared to HCs. We found a significant decrease in EGF levels among MDD patients (p=0.009). A substantial increase in DSM-5 score was also observed in MDD patients, as expected (p<0.001) …...” do not infer a correlation between HAM-D and EGF concentration. It is essential to conduct the correlation analysis itself, rather than interpreting the results that allegedly lead to the inference of correlation.

***Author responses***

*Thank you for your observation and suggestion. We have performed a correlation study and modified our manuscript (page 9, line 16-20).*

*You will find the correct statement in conclusion also (page 10, line 21-23).*

**Comment 3:**

From the statements in paragraph 4 “Furthermore, from sex-specific scatter plot graph where the distribution of serum EGF levels (pg/ml) against Ham-D score was depicted, it could be observed that the severity of depression was higher in females compared to males. After laboratory analysis, it was observed that the Ham-D score was higher in females than males, and the score difference between female subjects was higher than the score difference between male subjects. At the same time, serum EGF levels were lower in MDD females when compared to MDD males.”, it is clear that the authors have only made an observational interpretation. The statement that EGF causes depression more in women sounds ambitious on several counts. First, since the study design is cross-sectional, causality between variables cannot be established. The other is that the EGF concentration-severity of depression-gender relationship suggested by the authors can only be confirmed/falsified within an interaction model (e.g., factorial analysis of variance).

***Author responses***

*Thank you for your observation and suggestion. The independent sample t-test was performed between MDD male and MDD female subjects to find out the differences between EGF concentrations and HAM-D scores. But no significant differences were observed. We wanted to explore the variability among MDD males and females with a view to helping the scientific community in future. As per your suggestion we have modified our manuscript (page 9, lone 27-32).*

**Comment 4:**

Limitation: I would expect that the authors add to the limitations that the duration of depression that could potentially influence the levels of inflammatory parameters.

***Author responses***

*Thank you for your valuable suggestion. We have added the limitation as per your suggestion (page 10, line 13-14).*
